# NRG1 fusion in a French cohort of invasive mucinous lung adenocarcinoma

**DOI:** 10.1002/cam4.838

**Published:** 2016-10-21

**Authors:** Michaël Duruisseaux, Anne McLeer‐Florin, Martine Antoine, Sanaz Alavizadeh, Virginie Poulot, Roger Lacave, Nathalie Rabbe, Jacques Cadranel, Marie Wislez

**Affiliations:** ^1^ Sorbonne UniversitésUPMC University Paris 06GRC n°04, TheranoscanF‐75252ParisFrance; ^2^ Plateforme de Génétique Moléculaire des TumeursPôle de Biologie et Pathologie CHU Grenoble et INSERM U 823‐Institut A Bonniot‐Université J FourierGrenobleFrance; ^3^ AP‐HPHôpital TenonService d'Anatomie pathologiqueF‐75970ParisFrance; ^4^ AP‐HPHôpital TenonPlateforme de Génomique des Tumeurs SolidesF‐75970ParisFrance; ^5^ AP‐HPHôpital TenonService de PneumologieF‐75970ParisFrance

**Keywords:** FISH, invasive mucinous adenocarcinoma, lung adenocarcinoma, molecular oncology, NRG1

## Abstract

Invasive mucinous lung adenocarcinoma (IMA) is a rare subtype of lung adenocarcinoma with no effective treatment option in advanced disease. *KRAS* mutations occur in 28–87% of the cases. *NRG1* fusions were recently discovered in *KRAS*‐negative IMA cases and otherwise negative for known driver oncogenes and could represent an attractive therapeutic target. Published data suggest that *NRG1* fusions occur essentially in nonsmoking Asian women. From an IMA cohort of 25 French patients of known ethnicity, driver oncogenes *EGFR, KRAS, BRAF, ERBB2* mutations, and *ALK* and *ROS1* rearrangements presence were analyzed. In the IMA samples remaining negative for these driver oncogenes, an *NRG1* rearrangement detection was performed by FISH. A driver oncogene was identified in 14/25 IMA, namely 12 *KRAS* mutations (48%), one *ROS1* rearrangement (4%), and one *ALK* rearrangement (4%). The detection of *NRG1* rearrangement by FISH was conducted in the 11 pan‐negative IMA. One sample was *NRG1* FISH‐positive and 100% of the tumor nuclei analyzed were positive. This *NRG1*‐positive patient was a 61‐year‐old nonsmoking woman of Vietnamese ethnicity and was the sole patient of Asian ethnicity of the cohort. She died 6 months after the diagnosis with a pulmonary multifocal disease. *NRG1* FISH detection should be considered in patients with IMA pan‐negative for known driver oncogenes. These results might suggest that *NRG1* fusion is more frequent in IMA from Asian patient. Larger studies are needed.

## Introduction

Invasive mucinous adenocarcinoma (IMA) of the lung represents 2–10% of all lung adenocarcinomas (LUAD) 1, 2, 3. This histological subtype is considered as being one of the most malignant subtypes of LUAD, and is associated with a poor prognosis, probably due to frequent late‐stage diagnosis 1, 2, 3. Standard chemotherapy is the unique treatment option at advanced stages, as to date no effective targeted therapy has shown its effectiveness.

The most commonly found genetic alterations in IMA are *KRAS* mutations, with a prevalence of 28–87% of cases 4, 5, 6, 7, 8, 9, 10, 11, 12. Recently, recurrent *CD74‐NRG1* somatic gene fusions were discovered in IMA cases otherwise negative for known driver oncogenes (*EGFR*,* KRAS*,* BRAF*,* ERBB2*,* ALK*,* ROS1)* 13. NRG1 (neuregulin 1) is usually not expressed in normal lung and in LUAD, but *NRG1* fusions lead to NRG1 III‐b3 isoform expression in IMA. By means of an extracellular EGF‐like domain, NRG1 III‐b3 binds the extracellular domain of ERBB3, leading to heterodimerization of ERBB3 with ERBB2. The resulting activation of the downstream PI3K‐AKT and MAPK pathways promotes anchorage‐independent growth of LUAD cell lines. As ERBB2‐ERBB3 dimers and PI3K‐AKT and MAPK pathways could be targetable, *NRG1* fusions represent promising therapeutic targets 14. Indeed, *NRG1* fusion‐mediated signaling could be effectively suppressed in vitro by tyrosine kinase inhibitors such as lapatinib and afatinib approved for clinical use.

*CD74* is the most frequently found *NRG1* fusion partner, but novel *NRG1* partners have been described, such as *SLC3A2‐NRG1 and VAMP2‐NRG1* in two independent cohorts of IMA and *RBPMS‐NRG1*,* WRN‐NRG1,* and *SDC4‐NRG1* in a cohort of LUAD and squamous lung carcinomas 11, 12, 15.

*NRG1* fusions could drive 7–27% of IMA and published data suggest that these oncogenic fusions essentially occur in nonsmoking women of Asian origin 11, 13, 16. In this study, we sought to examine the prevalence and the clinical profile associated with *NRG1* fusions in a French cohort of IMA patients.

## Materials and Methods

### Population studied

Twenty‐five consecutive IMA patients surgically treated at Tenon Hospital (AP‐HP), France, from 1991 to 2013, were retrieved from the Chest department database. The diagnosis was confirmed by a lung cancer pathologist (MA) and was based on the 2015 WHO classification of tumors of the lung 1. Clinical findings at diagnosis and follow‐up data were recorded. All patients signed a research informed consent form, permitting analysis of their biological samples. This study was approved by our hospital's ethics human research committee.

### *EGFR, KRAS, BRAF,* and *ERBB2* mutation analyses

For each formalin‐fixed paraffin‐embedded (FFPE) specimen, a 3‐*μ*m tissue section was stained with H&S and examined by light microscopy to determine the percentage of tumor cells. After DNA isolation (QIAamp DNA mini kit^®^, Qiagen, Courtaboeuf, France) from three 20 *μ*m tissue sections, *EGFR* mutations G719S, T790M, and L858R (exons 18, 20, and 21, respectively), *KRAS* mutations G12S, G12R, G12C, G12A, G12V, and G13D (exon 2), and *BRAF* mutations V600E and V600K (exon 15) were detected with TaqMan^®^Assays (Custom TaqMan^®^ SNP Genotyping Assays, Life Technologies SAS, Saint Aubin, France). *EGFR* exon 19 deletions, *EGFR* exon 20 insertions and *ERBB2* exon 20 insertion were detected by sizing analysis. Sequencing data were then analyzed using SeqScape software.

### ALK and ROS1 immunohistochemistry

Immunostainings of the ALK and ROS1 proteins were performed on 3‐*μ*m tissue sec tions on a Benchmark Ventana staining module (Ventana^®^, Roche Diagnostics, Meylan, France) using a primary monoclonal ALK antibody (Clone 5A4, Ab 17127; Abcam, Paris, France) diluted at 1:50 for 2 h at 37°C, or a primary monoclonal ROS1 antibody (Clone D4D6, #3287, Cell Signaling Technology^®^, Danvers, MA) at a dilution of 1:50 2 h at 20°C, as previously described. Positive external controls were performed, using a LUAD specimen that had been previously validated for *ALK* rearrangement by fluorescent in situ hybridization and the *ROS1*‐rearranged cell line HCC78. The staining scores were assessed as follows: 0, no staining; 1+, faint cytoplasmic staining; 2+, moderate cytoplasmic staining; and 3+, intense granular cytoplasmic staining. The presence of 10% of cells stained with an intensity of ≥2 was considered as positive staining. Specimens with a positive staining score were tested for *ALK* or *ROS1* rearrangement by FISH.

### *ALK*,* ROS1,* and *NRG1* break‐apart FISH

FISH was performed on unstained 4‐*μ*m FFPE tumor‐tissue sections using an *ALK* break‐apart probe set (Vysis LSI *ALK* Dual Color^®^, Break Apart Rearrangement Probe; Abbott Molecular, Rungis, France) or a ZytoLight^®^ SPEC *ROS1* Dual Color Break Apart Probe (ZytoVision, Bremerhaven, Germany) and a paraffin‐pretreated reagent kit (Vysis^®^, Abbott Molecular) according to the manufacturer's instructions. Tumor tissues were considered *ALK*‐positive if >15% of the cells showed split orange and green signals and/or single orange signals or *ROS1*‐positive if >15% of the cells showed split orange and green signals and/or single green signals.

As *NRG1* fusions have been described in tumors without *EGFR*/*KRAS*/*BRAF*/*HER2* mutations and *ALK/ROS1* rearrangements, *NRG1* break‐apart FISH was performed only in pan wild‐type samples.

An *NRG1*‐specific fluorescent DNA probe was used kindly provided by ZytoVision (Zytolight SPEC NRG1 Dual Color Break Apart, ZytoVision, Bremerhaven, Germany). This probe contains green and orange‐labeled polynucleotides, which target sequences mapping in 8p12 proximal to the *NRG1* break point region. The 3' *NRG1* probe is labeled with an orange spectrum fluorophore and the 5' *NRG1* probe with a green spectrum fluorophore. The quality of each FISH experiment was categorized as good, moderate, or poor, according to the quality of the hybridization signals, and the presence of no to a very high fluorescent background noise, respectively. Tumor tissues were considered *NRG1* FISH‐positive when >15% of the nuclei harbored either a split pattern with 3′ and 5′ signals separated by a distance superior to the diameter of the largest signal, or isolated 3′ (orange) signals. This threshold was chosen by analogy with the threshold commonly used for other FISH assays for gene rearrangement detection in FFPE lung tumor samples, such as *ALK*,* ROS1,* or *RET* gene rearrangements.

Nuclei were counterstained with DAPI/Vectashield^®^ (Vektor Laboratories, Burlingame, CA) and were analyzed with a Leica CytoVision GSL10 FISH fluorescence capture system^®^ (Leica, Nanterre, France) under a 63x oil immersion objective. Signals were enumerated with the CytoVision imaging system^®^ (Leica). At least 100 nuclei were analyzed (mean = 126) for each tumor sample.

## Results

Clinical and molecular findings for the 25 IMA patients are shown in Table 1. All the driver oncogenes detected were mutually exclusive.

**Table 1:** Individual clinical and molecular characteristics of patients with invasive mucinous adenocarcinoma

Samples	Sex	Age	Ethny	Smoking (pack year)	Driver oncogene
1	F	78	Caucasian	Never	None
2	F	60	Caucasian	Never	None
3	M	62	Caucasian	Never	None
4	F	60	Caucasian	Never	None
5	M	47	North African	Ever	None
6	M	56	Caucasian	Ever	None
7	F	55	Caucasian	Never	None
8	F	68	Caucasian	Never	None
9	F	61	Asian	Never	*NRG1*
10	M	46	North African	Never	None
11	M	57	Caucasian	Ever	None
12	M	63	Caucasian	Ever	*KRAS*
13	M	87	Caucasian	Ever	*KRAS*
14	M	54	Caucasian	Ever	*KRAS*
15	M	58	Caucasian	Ever	*KRAS*
16	M	71	Caucasian	Ever	*KRAS*
17	F	77	Caucasian	Ever	*KRAS*
18	M	70	Caucasian	Ever	*KRAS*
19	M	69	Caucasian	Ever	*KRAS*
20	M	73	Caucasian	Ever	*KRAS*
21	F	58	Caucasian	Ever	*KRAS*
22	M	78	Caucasian	Ever	*KRAS*
23	M	78	Caucasian	Never	*KRAS*
24	F	55	Caucasian	Ever	*ALK*
25	F	82	Caucasian	Never	*ROS1*

**Figure 1:**
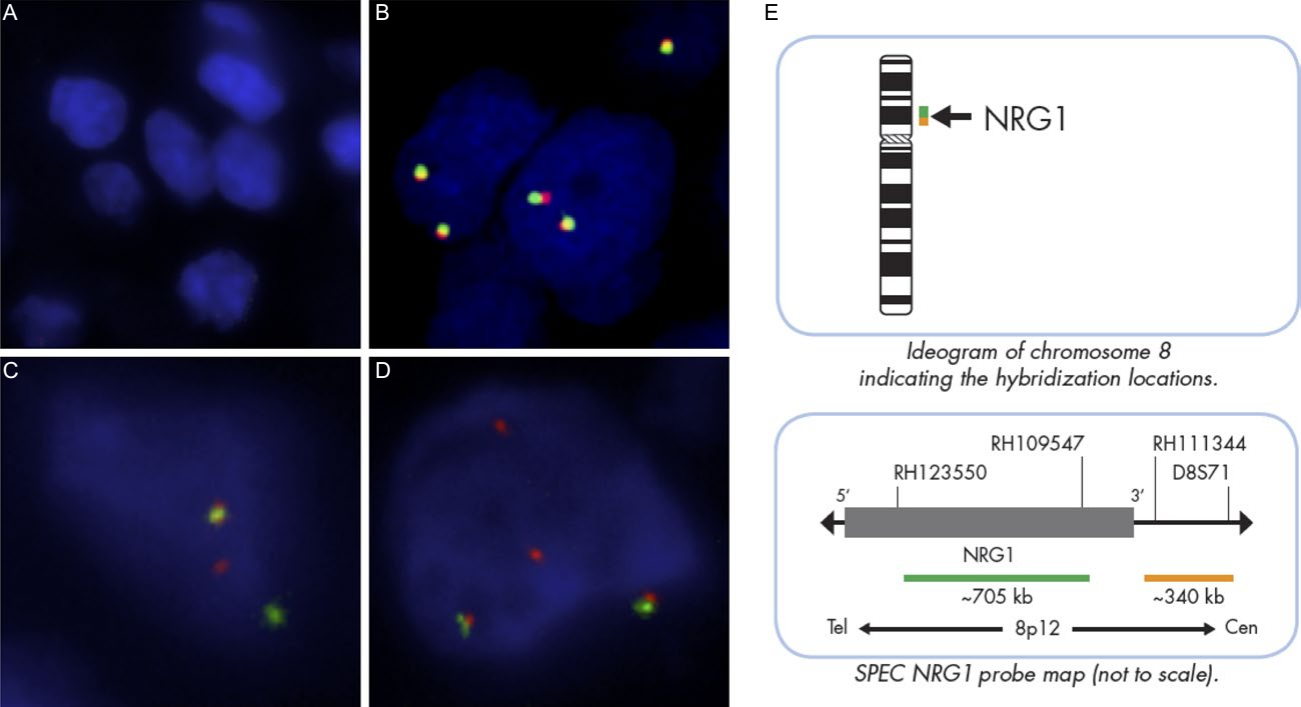
Patterns of *NRG1* FISH hybridization in our study (A) noninterpretable (absence of FISH signal), (B) negative with two fusion signals per nucleus, (C) negative with the presence of a split signal (one orange and one green signal) in <15% of the nuclei, (D) positive with at least one isolated orange signal in more than 15% of the nuclei. Original magnification ×630. (E) Ideogram of chromosome 8 and *NRG1* probe map for the ZytoLight^®^ SPEC NRG1 Dual Color Break‐apart Probe (ZytoVision), kindly provided by ZytoVision.

After analysis for *EGFR*,* KRAS*,* BRAF,* and *ERBB2* mutations and *ALK* and *ROS1* rearrangements, 11 samples remained wild‐type for all driver oncogenes and were analyzed for *NRG1* rearrangement by break‐apart FISH. The FISH patterns found in our cohort are depicted in Figure 1. The clinical findings and FISH quality and characteristics of each sample analyzed are shown in Table 2.

**Table 2:** Patient characteristics and *NRG1* FISH results in invasive mucinous adenocarcinoma tested for *NRG1* fusion

Samples	Date of samples conditioning	Sex	Age	Ethny	Smoking (pack year)	FISH results	Positives tumor cells (%)	Hybridation quality
1	1991	F	78	Caucasian	Never	NI	_	No FISH signal
2	2005	F	60	Caucasian	Never	Negative	1.0	Poor
3	1999	M	62	Caucasian	Never	NI		No FISH signal
4	2009	F	60	Caucasian	Never	Negative	1.0	Poor
5	2010	M	47	North African	Ever (40)	Negative	6.8	Moderate
6	1994	M	56	Caucasian	Ever (58)	NI		No FISH signal
7	2001	F	55	Caucasian	Never	NI		No FISH signal
8	2013	F	68	Caucasian	Never	Negative	7.4	Good
9	2006	F	61	Asian	Never	Positive	100	Good
10	2000	M	46	North African	Never	NI		No FISH signal
11	1995	M	57	Caucasian	Ever (65)	NI		No FISH signal

F, female; M, Male; NI, Not interpretable.

**Figure 2:**
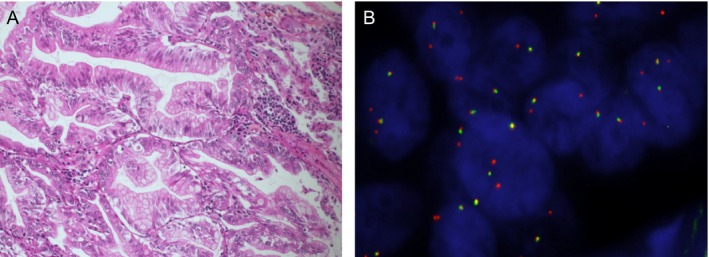
Representative histopathological features (A) and break‐apart *NRG1* FISH result (B) of the *NRG1*‐positive IMA case. (A) Goblet or columnar well differentiated tumoral cells with abundant intracytoplasmic mucin and small basally located nuclei (Hematoxylin‐Eosin‐Saffron, original magnification ×20) (B) Tumor nuclei hybridized with the ZytoLight^®^ SPEC NRG1 dual color beak‐apart probe (ZytoVision). All tumor cell nuclei analyzed were positive, showing at least one isolated 3' (orange) signal. Original magnification ×630.

**Figure 3:**
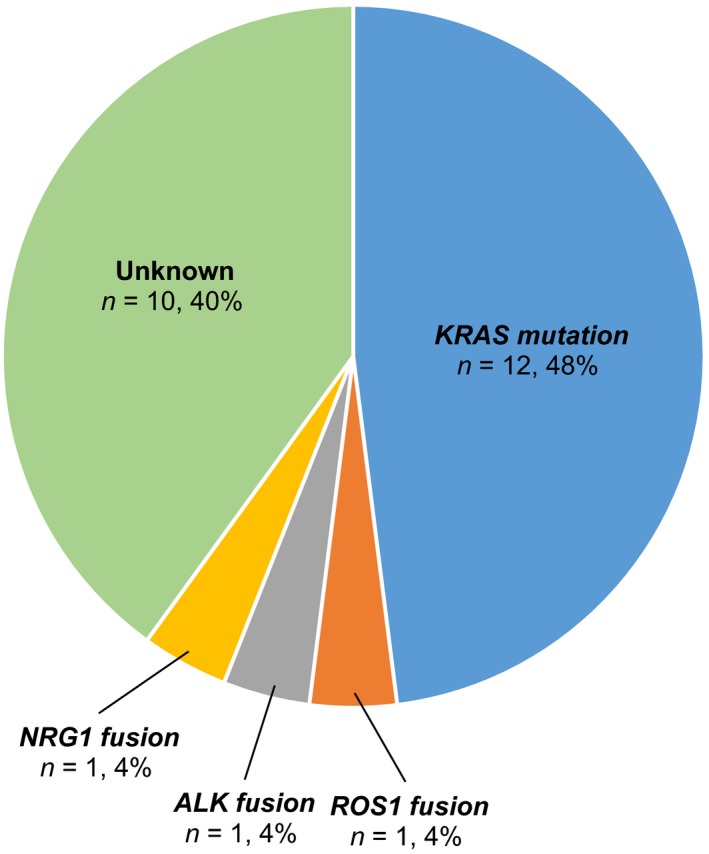
Pie chart of the frequencies of driver oncogenes detected. All driver oncogenes detected were mutually exclusive. Note that *NRG1* FISH was performed only in the 11 samples wild‐type for *EGFR*,*KRAS*,*ERBB2,* and *BRAF* mutations and *ALK* and *ROS1* rearrangements.

One sample was *NRG1* FISH‐positive and 100% of the tumor nuclei analyzed were positive, harboring at least one isolated orange signal, together with at least 1 fusion signal (Fig. 2). The frequency of each driver oncogene is shown in Figure 3.

This *NRG1*‐positive patient was a 61‐year‐old nonsmoking woman. She was born in Vietman to Vietnamese parents and migrated in France in 1976. She had a history of cured left breast cancer in 1988 treated with sequential neoadjuvant chemotherapy, radical mastectomy and chest wall irradiation, an ileal and pulmonary tuberculosis in 2003 successfully treated with antibiotics, a minimal change nephrotic syndrome requiring a daily corticosteroid treatment until 2004 and an insulin‐dependent diabetes. She presented with cough and dyspnea in April 2006. Chest computed tomography (CT) showed diffuse pulmonary parenchymal involvement with alveolar consolidation and pseudo nodules with peripheral ground‐glass opacities in the lower left lobe. The upper left lobe was destroyed by sequelae of tuberculosis. Diagnosis was obtained by bronchoscopic cytology. Abdominal CT, brain magnetic resonance imaging (MRI), and positron emission tomography using 18F‐fluorodeoxyglucose revealed no evidence of mediastinal node involvement or extra thoracic metastasis. Because of upper left lobe destruction, a left pneumonectomy was performed. Pathological analysis revealed an IMA which was TTF1 negative and CK7 and CK20 positive. The chest wall was invaded in an extent inferior to 1 centimeter and tumor cells were observed in one intralobar node. The tumor was classified as pT3N1M0. In view of the medical history of the patient, adjuvant chemotherapy was not administered and radiotherapy of the chest wall was performed. The disease relapsed 5 months after the surgery with appearance of numerous nodules in the remaining right lung on chest CT. The patient was enrolled in the IFCT‐0504 clinical trial evaluating erlotinib or carboplatin/paclitaxel in advanced lepidic adenocarcinoma and was randomized in the erlotinib arm. After 4 weeks of erlotinib, the patient presented a respiratory failure secondary to a nondocumented right interstitial lung disease (ILD) which could be related to a disease progression or an erlotinib‐induced ILD. She died after two weeks in intensive critical care unit.

## Discussion

*NRG1* rearrangements may be found by FISH in IMA wild‐type for *EGFR*,* KRAS*,* BRAF*,* ERBB2*,* ALK,* and *ROS1*. Our series of 25 IMA showed one *NRG1* FISH‐positive case, corresponding to a prevalence of 4%. Previous works using high throughput transcriptome sequencing in frozen samples or anchored multiplex PCR and next‐generation sequencing in FFPE samples estimated prevalence for *NRG1* fusions in IMA of 7–27% 11, 12, 13. The lower prevalence in our study could be due to the lesser sensitivity of FISH assay in FFPE. The FISH signals were of poor quality in 6/11 cases and corresponded to samples fixed prior to 2003 when preanalytical tissue handling steps were less standardized.

The *NRG1* FISH‐positive case was a Vietnamese nonsmoking woman, corresponding to the expected clinical profile reported in previous study (Table 3) 11, 13, 16. It is remarkable that the only *NRG1*‐positive case occurred in the sole patient of Asian ethnicity in our cohort. We speculate that *NRG1* fusions might occur at a lower prevalence in IMA from Caucasian patients. Shim et al. reported the molecular analysis of two cohorts of IMA, one from Caucasian patients (*n* = 31) and one form Asian patients (*n* = 41). A trend for a lower prevalence of fusion in Caucasian was found but type of fusion according to ethnicity was not given.

**Table 3:** Characteristics of published patients with *NRG1*‐positive invasive mucinous adenocarcinoma

	Sex	Age	Ethny	Smoking (pack year)	Gene fusion	Reference
1	Female	64	Caucasian	Never	*CD74‐NRG1*	Fernandez‐Cuesta et al. 13
2	Female	73	Asian	Never	*CD74‐NRG1*	Fernandez‐Cuesta et al. 13
3	Female	72	Asian	Never	*CD74‐NRG1*	Fernandez‐Cuesta et al. 13
4	Female	66	Asian	Never	*CD74‐NRG1*	Fernandez‐Cuesta et al. 13
5	Female	31	Asian	Never	*CD74‐NRG1*	Fernandez‐Cuesta et al. 13
6	Male	55	Asian	Ever (47)	*CD74‐NRG1*	Nakaoku et al. 11
7	Female	68	Asian	Never	*CD74‐NRG1*	Nakaoku et al. 11
8	Female	78	Asian	Never	*CD74‐NRG1*	Nakaoku et al. 11
9	Female	47	Asian	Never	*CD74‐NRG1*	Nakaoku et al. 11
10	Female	53	Asian	Never	*CD74‐NRG1*	Nakaoku et al. 11
11	Female	66	Asian	Never	SLC3A2‐NRG1	Nakaoku et al. 11
12	Female	89	Asian	Never	*CD74‐NRG1*	Gow et al. 16
13	Female	65	NA	Never	*CD74‐NRG1*	Shim et al. 12
14	Male	84	NA	Never	*CD74‐NRG1*	Shim et al. 12
15	Male	56	NA	Ever	*CD74‐NRG1*	Shim et al. 12
16	Female	73	NA	Never	*CD74‐NRG1*	Shim et al. 12
17	Female	58	NA	Never	*VAMP2‐NRG1*	Shim et al. 12
18	Female	62	Asian	Never	*NRG1* a	Duruisseaux et al. (this issue)

a Partner gene unknown.

The results of our study might indirectly suggest the scarcity of *NRG1* fusions in IMA in Caucasian patients. However, there is a need of a dedicated study to answer the question of whether or not the prevalence of *NRG1* fusion differs according to ethnicity. As *NRG1* fusions could be targetable, *NRG1* FISH detection should be considered in patients with IMA pan‐negative for *EGFR, KRAS, BRAF, ERBB2, ALK,* and *ROS1*.

## Conflict of Interest

None declared.
